# Oxidation of Isodrimeninol with PCC Yields Drimane Derivatives with Activity against *Candida* Yeast by Inhibition of Lanosterol 14-Alpha Demethylase

**DOI:** 10.3390/biom10081101

**Published:** 2020-07-24

**Authors:** Victor Marin, Andres Iturra, Andres Opazo, Bernd Schmidt, Matthias Heydenreich, Leandro Ortiz, Verónica A. Jiménez, Cristian Paz

**Affiliations:** 1 Laboratory of Natural Products & Drug Discovery, Department of Basic Science, Universidad de La Frontera, Av. Francisco Salazar 01145, 4780000 Temuco, Chile; v.marin03@ufromail.cl (V.M.); profesor.quimica.locz@gmail.com (A.I.); 2 Universidad de Concepción, Departamento de Microbiología, Laboratorio de Investigación en Agentes Antibacterianos (LIAA), Barrio Universitario S/N, 160-C 1807 Concepción, Chile; andopazo@udec.cl; 3 Universität Potsdam, Institut für Chemie, Karl-Liebknecht-Str. 24-25, D-14476 Potsdam, Germany; bernd.schmidt@uni-potsdam.de (B.S.); mheydenr@uni-potsdam.de (M.H.); 4 Universidad Austral de Chile, Instituto de Ciencias Química, Facultad de Ciencias, Universidad Austral de Chile, Casilla 567, 5091000 Valdivia, Chile; leandro.ortiz@uach.cl; 5 Universidad Andres Bello, Sede Concepción, Departamento de Ciencias Químicas, Facultad de Ciencias Exactas, Autopista Concepción-Talcahuano 7100, 4030000 Talcahuano, Chile

**Keywords:** Isodrimeninol, PCC oxidation, *Candida* yeast, molecular docking, lanosterol 14-alpha demethylase

## Abstract

*Candida* species cause an opportunistic yeast infection called Candidiasis, which is responsible for more than 50,000 deaths every year around the world. Effective treatments against candidiasis caused by non-albicans *Candida* species such as *C. glabrata, C. parapsilosis, C. aureus,* and *C.* *krusei* are limited due to severe resistance to conventional antifungal drugs. Natural drimane sesquiterpenoids have shown promising antifungal properties against *Candida* yeast and have emerged as valuable candidates for developing new candidiasis therapies. In this work, we isolated isodrimeninol (**C1**) from barks of *Drimys winteri* and used it as starting material for the hemi-synthesis of four sesquiterpenoids by oxidation with pyridinium chlorochromate (PCC). The structure of the products (**C2**, **C3**, **C4,** and **C5**) was elucidated by 1D and 2D NMR spectroscopy resulting in **C4** being a novel compound. Antifungal activity assays against *C. albicans, C. glabrata,* and *C. krusei* revealed that **C4** exhibited an increased activity (IC_50_ of 75 μg/mL) compared to **C1** (IC_50_ of 125 μg/mL) in all yeast strains. The antifungal activity of **C1** and **C4** was rationalized in terms of their capability to inhibit lanosterol 14-alpha demethylase using molecular docking, molecular dynamics simulations, and MM/GBSA binding free energy calculations. In silico analysis revealed that **C1** and **C4** bind to the outermost region of the catalytic site of 14-alpha demethylase and block the entrance of lanosterol (**LAN**) to the catalytic pocket. Binding free energy estimates suggested that **C4** forms a more stable complex with the enzyme than **C1**, in agreement with the experimental evidence. Based on this new approach it is possible to design new drimane-type sesquiterpenoids for the control of *Candida* species as inhibitors of 14-alpha demethylase.

## 1. Introduction

Candidiasis is a common opportunistic yeast infection caused by *Candida* species that affects more than 250,000 people and causes more than 50,000 deaths every year [1]. *Candida albicans* is the leading cause of candidemia, but other species such as *C. glabrata*, *C. parapsilosis*, *C. aureus,* and *C. krusei* are also relevant for the development of these diseases [2]. Four types of drug are in use for the treatment of *Candida* infections, namely azoles (fluconazole, itraconazole, voriconazole), polyenes (amphotericin B), echinocandins (anidulafungin, caspofungin, and micafungin), and the pyrimidine flucytosine, but only fluconazole and echinocandins are recommended as first-line agents for invasive candidiasis [3]. Fluconazole is the most widely prescribed antifungal agent. This compound inhibits the enzyme lanosterol-C14α-demethylase (CYP51) responsible for ergosterol biosynthesis by lanosterol demethylation [4]. Ergosterol is a critical steroidal component of the fungal cell membranes. Inhibition of ergosterol synthesis creates an accumulation of 14-methyl sterols, leading to a fungistatic effect instead of a fungicide action. Several non-*albicans Candida* species show resistance to antifungal drugs, for instance *Candida krusei* is intrinsically resistant to antifungals, whereas *Candida glabrata* acquires resistance after exposure to antifungal agents by the overexpression of multidrug transporters [5,6]. These undesired effects highlight the relevance of discovering novel antifungal agents against yeast infections caused by *Candida* species.

Natural compounds are a valuable source of antifungals and have been traditionally used by First Nations people for the treatment of diverse diseases. For example, the *Mapuche* people in Chile use barks and leaves of the tree *Drimys winteri* for the treatment of skin injuries. *Drimys winteri* is rich in bioactive drimane sesquiterpenoids such as polygodial, which is a 1,4-dialdehyde with potent antifungal activity against *Candida albicans* and other fungal strains [7], as well as trypanocidal [8] and insecticidal properties [9]. Polygodial has a minimum inhibitory concentration (MIC) of 3 μg/mL against *C. albicans* which is around three times stronger than fluconazole [10,11]. Its activity is potentiated by combination with miconazole or anetole [10,11]. The antifungal properties of polygodial have been attributed to the reactivity of dialdehydes to amines, forming a pyrrole moiety that causes a covalent interaction with proteins. This, together with the hydrophobic interaction, can disrupt the membrane by a nonionic surface-active action [12]. Despite its antifungal activity, polygodial has a pungent flavor due to activation of transient receptor potential channels (TRP) [13], together with irritant properties in eyes and soft skin, that make the use of polygodial unviable.

Drimane sesquiterpenoids share a common structural core with lanosterol in the first two rings, thus they could reach the catalytic pocket of the enzyme lanosterol-C14α-demethylase and interfere with its functional activity, preventing ergosterol biosynthesis. This hypothesis opens new possibilities for the discovery and optimization of novel antifungal agents targeting lanosterol-C14α-demethylase starting from natural drimane sesquiterpenoids obtained from traditional *Mapuche* medicine sources such as *Drimys winteri*. 

The phytochemical richness and pharmacological potential of *Drimys winteri* is remarkable. For example, bioactive sesquiterpene lactones such as cinnamolide and drimenin have been isolated from this source showing potent inhibitory activity against the human α4β2 nicotinic acetylcholine receptors that are involved in drug addiction [14]. Also, alcohols such as drimendiol have shown antibacterial quorum sensing activity [15,16] and they have been used as a scaffold for the hemisynthesis of nitrogenated derivatives with antifungal activity against *Candida albicans* [17]. In this work, we aim at obtaining novel drimane sesquiterpenoids from the partial oxidation of isodrimeninol (**C1**) isolated from barks of *Drimys winteri*. Our goal is to obtain hemi-synthetic derivatives with enhanced antifungal properties that can be used as novel molecular scaffolds to develop new drugs against Candida species.

Here, we describe the isolation of isodrimeninol (**C1**) from barks of *Drimys winteri* by preparative column chromatography on silica gel and exclusion chromatography on sephadex Lh-20. **C1** was partially oxidized by pyridinium chlorochromate (PCC) leading to four major products **C2**, **C3**, **C4**, and **C5**, which were fully elucidated by 1D and 2D NMR spectroscopic methods. In vitro antifungal activity of compounds **C1**–**C5** was assessed in terms of their minimum inhibitory concentration (MIC) against *C. albicans, C. glabrata* and *C. krusei*. Our results revealed that **C4** (IC_50_ of 75 μg/mL) exhibits an increased antifungal performance compared to **C1** (IC_50_ of 125 μg/mL), whereas **C2**, **C3**, and **C5** were inactive at concentrations lower than 200 μg/mL. The potential interaction of **C4** and **C1** drimane sesquiterpenoids with lanosterol-C14α-demethylase was investigated by molecular modelling methods such as molecular docking, molecular dynamics simulations and MM/GBSA binding free energy calculations. Molecular modelling suggests that **C1** and **C4** bind to the outermost region of the catalytic site of lanosterol 14-alpha demethylase in such a way that they block the entrance of lanosterol to the catalytic pocket. In silico analysis revealed that **C4** exhibits a higher affinity toward the binding pocket compared to **C1**, in agreement with the experimental observations concerning their antifungal activity. 

## 2. Materials and Methods 

### 2.1. General Information

Analytical thin-layer chromatography (TLC) was carried out on Merck Silica Gel 60F254 sheets (Darmstadt, Germany). It was employed for monitoring the reaction progress and purification of the newly synthesized compounds, using an elution mixture of n-hexane: ethyl acetate = 9:1 and UV light (254 nm) together with molybdophosphoric acid and heating for visualization. Preparative chromatography was performed using Merck silica gel 60 and Sephadex LH-20 (25−100 μm; Aldrich, Santiago, Chile). Solvents and fractions were concentrated in a Büchi R100 rotavap. Solvents used in this study were distilled prior to use and dried over appropriate drying agents [18].

### 2.2. Purification of Isodrimeninol from Drimys Winteri

The purification of isodrimeninol (**C1**) was done by preparative column chromatography from a sub-fraction of *Drimys winteri*, previously extracted from barks of the tree macerated with ethyl acetate (EtOAc), which was kept at −80 °C with the code F5 [14]. In this work, the fraction F5 was further purified by Silica gel column chromatography. The compound eluted with a mixture of solvents at 4:1 hexane/EtOAc giving a concentrate rich in **C1** together with the lignan sesamin. The mixture was once more eluted though sephadex Lh-20 with isopropanol as eluent to give a clear oil identified as **C1** (isodrimeninol, 5.56 g, colorless oil, 0.059% yield). 

### 2.3. Oxidation of C1 with Pyridinium Chlorochromate (PCC)

To a solution of **C1** (236 g/mol, 0.424 mmol) in dichloromethane (20 mL) was added dropwise 1 equivalent of PCC (215.5 g/mol, 91.4 mg). Then, the reaction mixture was stirred at room temperature under nitrogen atmosphere for 6 h. The solvent was removed under vacuum and the gummy residue was further purified by silica gel column chromatography with hexane/EtOAc (9:1 *v*/*v*). The products were obtained in the following order: **C2** (232 g/mol, 19.5 mg, 20% yield), **C3** (234 g/mol, 9.8 mg, 10% yield), **C4** (250 g/mol, 40.3 mg, 38% yield), **C5** (248 g/mol, 21.0 mg, 20% yield).

### 2.4. Structural Identification of Compounds

The structures of compounds **C1**–**C5** were elucidated by 1D and 2D NMR. The ^1^H- and ^13^C NMR spectra were recorded in CDCl_3_ solution in 5 mm tubes at RT on a Bruker Avance III 600 MHz spectrometer (Bruker Biospin GmbH, Rheinstetten, Germany) at 600.13 (^1^H) and 150.61 (^13^C) MHz, with the deuterium signal of the solvent as the lock and TMS (for ^1^H) or the solvent (for ^13^C) as internal standard. All spectra (^1^H, ^13^C, gs-H,H−COSY, edited HSQC, and gs-HMBC) were acquired and processed with the standard Bruker software. 

### 2.5. Antifungal Assays against Candida Species 

Yeast strains *C. albicans* ATCC 90028, *C.* glabrata ATCC 90,030 and *C. krusei* ATCC 6258 were acquired from the Laboratorio de Investigación en Agentes Antibacterianos (LIAA), Departamento de Microbiología, Universidad de Concepción. The in vitro antifungal assay was performed in triplicate by using the microdilution broth method in 96-well microplates according to the recommendations of the Clinical and Laboratory Standards Institute [CLSI 2018]. Briefly, samples were dissolved in dimethyl sulfoxide (DMSO) at 1 mg/mL, followed by dilution in culture media (RPMI) to achieve concentrations from 200 to 1 μg/mL. The inoculum was adjusted to yield cell concentration of 2.5 × 10^3^ UFC/mL, the final DMSO content was 5% (*v*/*v*). One inoculated well was included as microorganism growth control. One non-inoculated well was included to ensure medium sterility. Fluconazole was used as a positive control at concentrations of 100, 75, 50, 25, 12, 8, and 4 μg/mL. Microplates were incubated at 37 °C for 48 h. The MIC values were determined in triplicate, at 24 h, as the lowest concentrations of each compound capable of inhibiting microorganism growth by visual inspection, compared to growth control.

### 2.6. Simulated Systems

The protein and **LAN** coordinates for **LAN** in complex with Saccharomyces cerevisiae lanosterol 14-alpha demethylase were retrieved from the crystallographic model with protein data bank PDB code 4LXJ. This structure was obtained from X-ray data with 1.90 Å resolution. Before simulation, the protein structure was checked to fix protein discontinuities considering the UNIPROT code P10614 and protonation states were set to pH 6.5 using the H++ web server [19,20]. The initial coordinates for compound **C1** were retrieved from the ZINC database under the code ZINC15148130 [21]. The structure of compound **C1** was employed as template to manually build the coordinates of compound **C4** using the Molefacture Plugin V1.5 implemented in the Visual Molecular Dynamics (VMD) software [22].

### 2.7. Molecular Docking Calculations

Blind protein–ligand molecular docking calculations were carried out to obtain the initial guess structures for the 14-alpha demethylase complexes with compounds **C1** and **C4**. To this aim the CB-Dock web server for cavity detection-guided protein–ligand blind docking calculations was employed [23]. CB-Dock predicts binding sites of a given protein, calculates the centers and sizes with a novel curvature-based cavity detection approach, and performs ligand docking with Autodock Vina [24]. The conformation with the highest rank according to docking score was selected to obtain equilibrated structures of the 14-alpha demethylase complexes using molecular dynamics simulations. 

### 2.8. Molecular Dynamics Simulations

Fully atomistic molecular dynamics (MD) simulations on 14-alpha demethylase complexes with compounds **C1** and **C4** were carried out to retrieve information regarding the possible molecular mechanism of action of these ligands as antifungal agents. Additionally, MD simulations were carried out in the ligand free protein and in the lanosterol (**LAN**) complex with 14-alpha demethylase as reference systems. Protein structure parameters were retrieved from the ff14SB force field. Simulation parameters for **C1**, **C4** and **LAN** consistent with the GAFF force field were obtained using the ANTECHAMBER module in the AMBER16 software with AM1-BCC atomic charges 25. Simulated systems were solvated in a cubic box of 10 Å length using a TIP3P explicit water model, adding Cl^-^ ions to maintain charge neutrality. MD simulations were performed using the pmemd.cuda program implemented in the AMBER16 software. MD protocol consisted of: (a) 1500 steepest descent minimization steps followed by 3500 conjugate gradient minimization steps for water molecules relaxation, (b) 1500 steepest descent minimization steps followed by 6500 conjugate gradient minimization steps for the entire system, (c) 500 ps of progressive NVT heating from 0 to 300 K (d) 500 ps of NVT equilibrium at 300 K with restrains applied to protein backbone, (e) 20 ns of NVT equilibrium at 300 K, and finally (f) 150 ns of unrestrained NPT production dynamics at 300 K and 1 bar from which production data were collected. During MD simulations, the cutoff for non-bonded terms was 10 Å, long-range electrostatics were treated using the particle-mesh Ewald approach, and the SHAKE algorithm was employed to constrain all bonds involving hydrogen [25,26].

### 2.9. Trajectory Analysis

The last 50 ns of MD trajectories were used to retrieve structural and energetic information regarding the association of **C1**, **C4**, and **LAN** to 14-alpha demethylase. Trajectory alignment and root-mean-square deviation (RMSD) for Cα atoms were carried out using MDLovoFit functionalities [27]. MM/GBSA binding free energy calculations were carried out to estimate the strength of protein–ligand interactions using the MMPBSA.py module in Amber16, under a single trajectory approach [27]. GB calculations were carried out using the modified GB model (igb = 5) with mbondi2, and α, β, and γ values of 1.0, 0.8, and 4.85, respectively. Dielectric constants for the solvent and the protein were set to 80 and 1, respectively. A salt concentration of 0.15 mol L^−1^ was considered in this process to mimic physiological conditions for binding free energy estimates. The entropic term was not included in our calculations due to the size of the systems under study. 

## 3. Results

### 3.1. Hemisynthetic Compounds by Oxidation of Isodrimeninol 

**Scheme 1:**
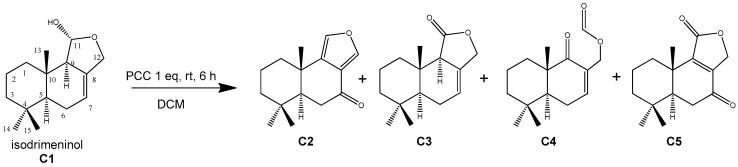
Molecular structures of drimane derivatives obtained by PCC oxidation of isodrimeninol.

The purification of the fraction F5 gave the drimane sesquiterpenoid isodrimeninol (**C1**) and the furofuran lignan sesamin. Oxidation of **C1** with PCC furnished four products **C2**–**C5**. The structures of each compound are given in Scheme 1.

Compounds **C2**, **C3**, **C4**, and **C5** were characterized by 1D and 2D NMR spectroscopy. ^1^H-NMR and ^13^C-NMR results are summarized in the Table 1 and Table 2. 

**C1**, (1R,5aS,9aS,9bR)-6,6,9a-trimethyl-1,3,5,5a,6,7,8,9,9a,9b-decahydronaphtho [1 ,2-c]furan-1-ol), also called isodrimeninol, is an 11*R*-configured hemiacetal, while compound **C2** is a furan obtained in a 20% yield, ((5aS,9aS)-6,6,9a-trimethyl-5a,6,7,8,9,9a-hexahydronaphtho[1,2-c]furan-4(5H)-one). Compound **C3**, (5aS,9aS,9bR)-6,6,9a-trimethyl-5,5a,6,7,8,9,9a,9b-octahydronaphtho[1,2-c]furan-1(3H)-one), also called drimenin, was purified by column chromatography in 10% yield after crystallization. **C4,** ((4aS,8aS)-5,5,8a-trimethyl-1-oxo-1,4,4a,5,6,7,8,8a-octahydronaphthalen-2-yl)methyl formate) was obtained in 38% yield, while compound **C5**, (7-ketoisodrimenine, (5aS,9aS)-6,6,9a-trimethyl-5a,6,7,8,9,9a-hexahydronaphtho[1,2-c]furan-1,4(3H,5H)-dione) was purified in 20% yield.

### 3.2. Anti-Candida Activity Assay

The broth microdilution method was employed for the determination of MIC values. Stock solutions (1 mg/mL) were prepared by dissolving the tested compounds and the reference antifungal drug, fluconazole, in sterile DMSO. The obtained values are presented in Table 3.

### 3.3. In Silico Assay

**Figure 1:**
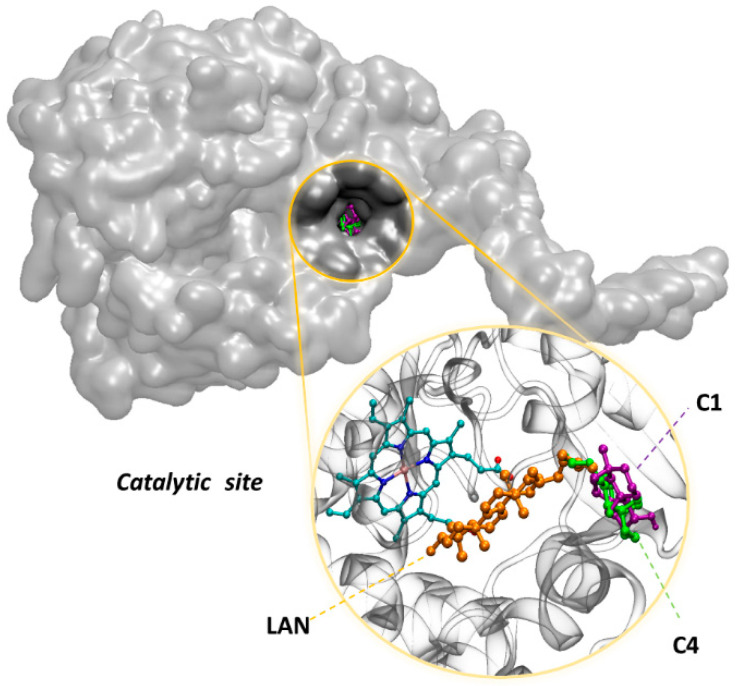
Best ranked binding poses for the association of **C1** (purple) and **C4** (green) to 14-alpha demethylase obtained from protein-ligand docking calculations with the CB-dock server. Structural superposition between the 14-alpha demethylase complexes with **C1** (purple), **C4** (green) and **LAN** (orange) obtained protein–ligand docking calculations.

Protein–ligand docking, and fully atomistic MD simulations were carried out to examine the molecular details of the association of 14-alpha demethylase complexes with compounds **C1** and **C4** as a molecular level approach to support the in vitro antifungal activity of these ligands. The structure of the **LAN** complex with 14-alpha demethylase was considered as a reference model for comparative purposes. Protein-ligand docking was carried out with the entire protein structure using the automated CB-Dock server. Blind docking was carried out to detect the suitable binding sites for each ligand followed by an automatic adjustment of cavity center and docking box size. A docking box size of 34 × 24 × 18 Å^3^ centered in the **LAN** binding cavity was chosen to conduct the ligand-protein protocol resulting in a series of bound poses for each compound. The best ranked poses for **C1** and **C4** are displayed in Figure 1.

**Figure 2:**
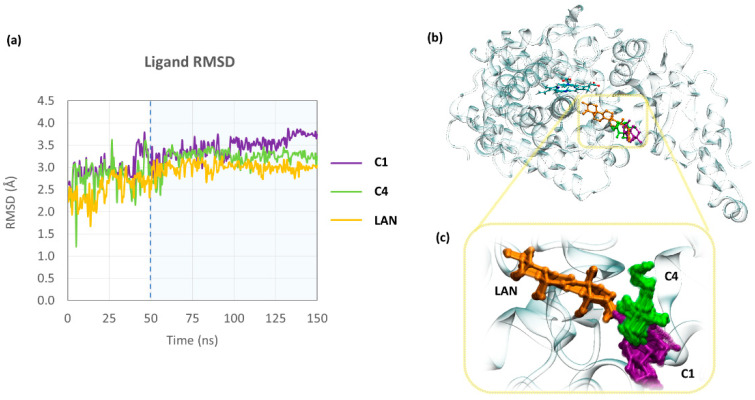
(**a**) Ligand RMSD (Å) calculations for 14-alpha demethylase complexes with **C1** (purple), **C4** (green) and **LAN** (orange) obtained from protein-aligned trajectories during the last 50 ns out of 150 ns MD simulations. (**b**) Representative equilibrated structures for 14-alpha demethylase complexes with **C1** (purple), **C4** (green) and **LAN** (orange) obtained from the analysis of 50 ns out of 150 ns MD trajectories. (**c**) Time evolution of the binding poses of **C1** (purple), **C4** (green) and **LAN** (orange) in complex with 14-alpha demethylase during the last 50 ns out of 150 ns MD trajectories.

The best ranked docked structures for 14-alpha demethylase complexes with **C1** and **C4** were subjected to 150 unrestrained MD simulations to examine the structural and energetic details related to their association to the target protein. MD trajectory analysis was carried out to verify the temporal stability of the docking predictions and to retrieve equilibrated structural and energetic information regarding the relative affinity of these ligands toward the target enzyme. Ligand RMSD plots for protein-aligned trajectories revealed that all systems reached equilibrium after the first 50 ns of MD simulations (Figure 2a). According to MD equilibrated trajectories, both compounds showed ability to block the entrance of the catalytic substrate to the reactive domain of the enzyme (Figure 2b). Time evolution plots for the binding poses of **C1**, **C4,** and **LAN** indicate that all ligands adopt a fixed location within the 14-alpha demethylase complexes with very small displacements in their binding poses along the simulation run (Figure 2c).

**Figure 3:**
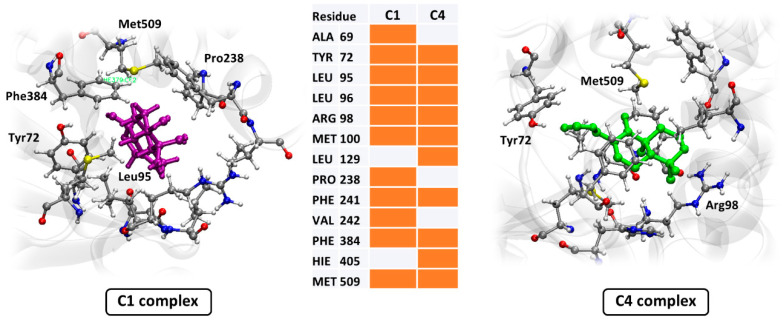
Binding site residues for **C1** (purple) and **C4** (green) complexes with 14-alpha demethylase predicted from the analysis of the last 50 ns out of 150 ns MD simulations. Common binding site residues are highlighted in the chart.

Trajectory analysis during the last 50 ns out of 150 ns MD runs was employed to identify the protein residues responsible for **C1** and **C4** association to 14-alpha demethylase, Figure 3. Our results revealed that both compounds exhibited similar interaction patterns with the **LAN** binding site with predominance of hydrophobic residues of the binding pocket. Common binding site residues are Tyr72, Leu95, Leu96, Arg98, Met100, Phe241, Phe384, and Met509.

MM/GBSA binding free energy calculations were carried out to obtain a quantitative estimate of the strength of the intermolecular association in **C1** (purple) and **C4** (green) complexes with 14-alpha demethylase. Binding free energy estimates are reported in Table 4 and account for an enhanced affinity of **C4** toward the binding cavity in the target protein compared to **C1**, in agreement with the results of our in vitro studies.

## 4. Discussion

Isodrimeninol, **C1,** was first isolated from *Polygonum hydropiper* [28] and later from the tree *Drimys winteri* [29]. This compound is also accessible in 11 steps from zamoranic acid [30]. Its oxidation with 1 equivalent of PCC for 6 h in dichloromethane at room temperature produced four compounds identified by 1D and 2D NMR and summarized in Scheme 1 and Table 1 and Table 2. The compound **C2** was previously synthesized from a manool derived diene in a multiple step synthesis [31] and from a sclareol derived diene in four steps, involving unselective photooxygenation reactions [32]. Analytical data obtained by us match those reported in the literature. A plausible pathway for the formation of **C2** involves allylic oxidation with double bond migration and elimination of water, which is facilitated by the well-known slightly acidic character of the PCC reagent [33]. Compound **C3**, also called drimenin, an annellated β-alkylidene-γ-lactone, was previously isolated from *Drimys winteri* [14], but has also been synthesized from racemic albicanol via lipase catalyzed kinetic resolution in a multiple step approach [34]. Analytical data obtained by us match those previously reported. The compound **C4** has a decalinone skeleton and is the main product of the PCC oxidation of isodrimeninol, accounting for 38% of the total yield of oxidation products. This compound has not been described in the literature, but it presumably results from an acid catalyzed elimination of water from the hemiacetal structure of **C1**, followed by oxidative cleavage of the C-C-double bond of the newly formed enol ether. Precedence for the oxidative cleavage of enol ether double bonds exists in the literature [35]. Compound **C5** is a 1,4-dicarbonyl compound with a keto group at position 7. It was first isolated from *Porella cordeana* [36], but has also been synthesized from the same manool derived diene as compound **C2 [31]**. Our analytical data agree with previously reported information for this compound.

The activity of synthetized compounds was evaluated in terms of the minimum inhibitory concentration values (MIC, μg/mL) against three *Candidas* yeast: *C. albicans* ATCC 90028; *C. glabrata* ATCC 90,030 and *C. krusei* ATCC 6258 by the broth microdilution method. Our results reveal that **C1** and **C4** show MIC values lower than 200 μg/mL, while **C4** showed the highest activity for the three strains assayed in this work, with a MIC value of 75 μg/mL. its activity against *C. krusei* is comparable with fluconazole. Protein–ligand docking predicted that **C1** and **C4** bind to the outermost region of the catalytic site of 14-alpha demethylase in such a way that they are able to block the entrance of any substrate to the catalytic pocket (Figure 1). According to protein–ligand docking calculations, the bound structures of **C1** and **C4** partially overlap with the aliphatic region of bound **LAN** in the 4LXJ crystallographic model as revealed by the structural superposition of the binding poses predicted for **C1** and **C4** and the crystallographic model for 14-alpha demethylase-**LAN** complex. MD simulations and ligand RMSD plots for protein-aligned trajectories revealed that all systems reached equilibrium after the first 50 ns of MD simulations (Figure 2a). Representative equilibrated structures for the 14-alpha demethylase complexes with **C1**, **C4,** and **LAN** confirmed that these ligands adopt binding poses that block the entrance of the catalytic substrate to the reactive domain of the enzyme (Figure 2b). Time evolution plots for the binding poses of **C1**, **C4,** and **LAN** indicate that all ligands keep a fixed location within the 14-alpha demethylase complexes with very small displacements in their binding poses along the simulation run (Figure 2c). MD simulations revealed that both ligands share a common binding pocket that mostly composed by hydrophobic protein residues (Figure 3), thus suggesting the predominance of van der Waals and hydrophobic interactions as main driving forces for the intermolecular association of **C1** and **C4** with 14-alpha demethylase. Binding free energy estimates predicted an enhanced affinity of **C4** toward the binding cavity in the target protein compared to **C1**, which is an auspicious result concerning the structural hypothesis that underlies this work. In this regard, the improved antifungal activity observed for **C4** could be related to a strengthened binding to 14-alpha demethylase. Neither **C1** nor **C4** were predicted to have a higher binding affinity toward the enzyme compared to **LAN**, which can be attributed to the significantly larger molecular size and hydrophobic surface of this latter ligand compared with **C1** and **C4**. Still, binding free energy estimates obtained for **C1** and **C4** association to 14-alpha demethylase suggest that both ligands act by forming stable complexes that block the entrance of the natural substrate leading to an inhibition of the enzymatic activity. The structural hypothesis that relates the antifungal activity of **C1** and **C4** with their association to the active site of 14-alpha demethylase is supported by extensive related experimental information showing the relationship between the inhibition of 14-alpha demethylase and the antifungal activity of ligands targeting this protein, such in the case of azole drugs [37]. However, no information is available about the inhibition of this key enzyme by related bicyclic natural compounds.

## 5. Conclusions

Candidiasis is the infection caused by the yeast Candida, among them *Candida albicans* is the most prevalent species. This infection can affect cutaneous, mucosal, and deep-seated organs, and in many developed countries, *Candida* spp. rank in the top three or four pathogens causing health-care-associated bloodstream infections [1]. Candidiasis caused by non-*albicans Candida* have a remarkable geographic variation. For instance, resistance in *C. glabrata* is more common in North America but was not detected in Asia-Pacific and Latin America, and *C. parapsilosis* with resistance to fluconazole was noted in Europe, but it was not detected in Asia-Pacific or Latin America, while *Candida krusei* resistance to azole have been observed only in North America [38].

The use of fluconazole as the first drug used in antifungal therapy has led to the development of resistance in Candida species emerging a new global concern, the non-albicans Candida resistance to drugs [39]. Drimane sesquiterpenoids have been an important source of drugs, among them polygodial, which displays a potent activity against many fungal strains. For instance, against *C. albicans* polygodial displays a MIC of 3 μg/mL, which is around three times stronger than fluconazole [10,11], but in spite of its antifungal activity polygodial has a pungent flavor due to activation of TRP channels, together with irritant properties in eyes and soft skin, that make the use of polygodial unviable. Its activity has been attributed to the reactivity of dialdehydes to amines, forming a pyrrole moiety that causes a covalent interaction with proteins. This, together with the hydrophobic interaction, can disrupt the membrane by a nonionic surface-active action. Here, we have shown that the drimane sesquiterpenoid moiety is similar to the first two cycles of lanosterol, then the drimane could also interact with the enzyme 14-alpha demethylase. In order to prove this idea, we have purified isodrimeninol from *Drimys winteri* which was used as a starting material for the synthesis of four new sesquiterpenoids, which revealed important differences in their structures, such as the activation of position 7 by a keto moiety in **C2** and **C5**, the non-conjugated lactone **C3,** and cleavage of the third cycle in **C4**. The antifungal potential for the five compounds was evaluated in vitro against three pathogenic Candida strains in terms of MIC. The results show that **C4**, a hitherto unknown compound, displays better antifungal activity than the other compounds against the three Candida strains, with the same MIC of 75 μg/mL. Apparently the five membered ring decreases the inhibitory activity of the molecule. Further in silico studies were performed toward fungal lanosterol-C14α-demethylase (CYP51), with the objective to investigate the molecular mechanism of action of the synthesized compounds. The molecular docking study and molecular dynamics suggest that **C1** and **C4** bind almost in the same position to the enzyme in such a way, that they block the entrance of lanosterol to the catalytic pocket with a good free binding energy toward the target enzyme of −25.7 and −30.7 kcal mol^−1^, respectively. The suggestion that **C4** displays a more stable complex with the enzyme is in accord with the inhibitory activity in vitro, where **C4** has an IC_50_ value of 75 μg/mL, compared to 125 μg/mL for **C1**. In spite of the moderate activity of **C4**, this new evidence makes it possible to design new drimane-type sesquiterpenoids for the control of Candida yeast by inhibition of the synthesis of ergosterol.

## Figures and Tables

**Table 1:** ^1^H-NMR (600 MHz, in CDCl_3._*δ* in ppm. *J* in Hz) results for sesquiterpenoids.

H	C1	C2	C3	C4	C5
1	1.24 (1H, *m*) 1.79 (1H, *ddd,* 13.1, 5.0, 2.8)	1.56 (1H, *m*) 2.07 (1H, *m*)	1.22 (1H, *m*) 2.49 (1H, *dq,* 13.4, 2.7)	1.36 (1H, *m*) 1.89 (1H, *m*)	1.33 (1H, *ddd*, 13.5, 13.5, 3.8) 2.65 (1H, *m*)
2	1.46 (1H, *m*) 1.59 (1H, *m*)	1.58 (1H, *m*) 1.71 (1H, *m*)	1.49 (1H, *m*) 1.59 (1H, *dq*, 13.7, 3.2)	1.58 (2H, *m*)	1.60 (1H, *m*) 1.70 (1H, *m*)
3	1.22 (1H, *m*) 1.46 (1H, *m*)	1.25 (1H, *m*) 1.51 (1H, *m*)	1.49 (1H, *m*) 1.24 (1H, *m*)	1.18 (1H, *m*) 1.45 (1H, *m*)	1.25 (1H, *dd*, 13.3, 4.1) 1.52 (1H*, m*)
5	1.30 (1H, *dd*, 11.7, 5.4)	1.78 (1H, *dd*, 13.4, 3.6)	1.36 (1H, *dd*, 11.7,5.3)	1.63 (1H, *dd*, 11.4, 4.4)	1.87 (1H, *dd*, 14.3, 3.0)
6	1.91 (1H, *m*) 2.14 (1H, *m*)	2.49 (1H, *dd*, 18.0, 13.4) 2.57 (1H*, ddd*, 18.0, 3.6, 0.5)	2.21 (1H, *m*) 1.98 (1H, *m*)	2.33 (1H, *dddd*, 19.6, 11.4, 3.9, 2.0) 2.44 (1H, *ddd*, 19.6, 5.0, 5.0)	2.50 (1H, *dd*, 17.5, 14.3) 2.63 (1H, *dd*, 17.3, 3.3)
7	5.51 (1H, *m*)	-	5.74 (1H, *br*, *s*)	6.98 (1H, *d*, 5.8)	-
9	2.20 (1H, *m*)	-	2.78 (1H, *br*, *s*)	-	-
11	5.28 (1H, *t*, 3.9) 11-OH: 3.29 (1H, *d*, 4.1)	7.91 (1H, *d,* 1.5)	-	8.08 (1H, *s*)	-
12	4.18 (1H, *dddd*, 11.3, 3.1, 1.5, 1.5) 4.48 (1H, *ddddd*, 11.3, 3.0, 3.0, 2.0, 2.0)	7.17 (1H, *d*, 1.5)	4.66 (2H, *m*)	4.76 (1H, *d*, 12.7) 4.83 (1H, *d*, 12.7)	4.82 (2H, *s*)
13	0.82 (3H, *s*)	1.25 (3H, *d*, 0.6)	0.90 (3H, *s*)	1.06 (3H, d, 0.6)	1.28 (3H, *s*)
14	0.92 (3H, *s*)	0.96 (3H, *s*)	0.92 (3H, *s*)	0.99 (3H, *s*)	0.96 (3H, *s*)
15	0.88 (3H, *s*)	0.92 (3H*, s*)	0.88 (3H, *s*)	0.91 (3H, *s*)	0.92 (3H, *s*)

**Table 2:** ^13^C-NMR (150 MHz, CDCl3. δ in ppm) results for sesquiterpenoids.

C	C1	C2	C3	C4	C5
1	40.0	38.3	38.4	33.1	33.2^b^
2	18.6	18.7	18.2	18.2	18.0
3	42.5	41.7	42.3	41.7	41.2
4	33.1	33.3^a^	33.0	33.8	33.3^b^
5	50.0	51.3	49.6	49.1	52.1
6	23.8	37.3	23.3	24.6	36.2
7	117.3	196.4	121.2	147.7	196.5
8	136.6	123.3	129.8	131.6	149.2
9	61.6	138.7	53.6	204.0	152.6
10	33.5	33.9	34.3	45.3	36.8
11	99.5	144.2	175.4	160.9	171.0
12	69.1	136.0	69.8	61.6	67.4
13	14.2	23.3	33.0	17.2	18.2
14	21.6	21.5	21.4	22.4	21.1
15	33.3	33.1^a^	13.9	32.4	32.9

a, b; or reversed.

**Table 3:** The minimum inhibitory concentration values (MIC, μg/mL) of the compounds **C1-C4**, n = 3.

Compound	*C. albicans* ATCC 90028	*C. glabrata* ATCC 90030	*C. krusei* ATCC 6258
**C1**	125	125	125
**C2**	>200	>200	>200
**C3**	>200	>200	>200
**C4**	75	75	75
**C5**	>200	>200	>200
Fluconazole	8	50	75

**Table 4:** MM/GBSA binding free energy estimates for the association of **C1**, **C4** and **LAN** to 14-alpha demethylase. Data was obtained from 1000 frames retrieved from the last 50 ns out of 150 ns MD trajectories. Data is reported as average ± standard error of the mean in kcal mol^−1^.

Ligand	Binding Free Energy Terms (kcal mol^−1^)				
	E_VdW_	E_el_	E_GB_	E_surf_	∆G_bind_
**LAN**	−60.8 ± 0.2	−7.0 ± 0.2	20.0 ± 0.1	−7.3 ± 0.1	−55.1 ± 0.2
**C1**	−30.9 ± 0.1	−1.9 ± 0.2	11.1 ± 0.1	−3.9 ± 0.1	−25.7 ± 0.2
**C4**	−36.1 ± 0.1	−3.4 ± 0.2	13.7 ± 0.1	−4.9 ± 0.1	−30.7 ± 0.2
